# Synthesis, characterization and toxicity studies of pyridinecarboxaldehydes and L-tryptophan derived Schiff bases and corresponding copper (II) complexes

**DOI:** 10.12688/f1000research.9226.1

**Published:** 2016-08-05

**Authors:** Margarita Malakyan, Nelly Babayan, Ruzanna Grigoryan, Natalya Sarkisyan, Vahan Tonoyan, Davit Tadevosyan, Vladimir Matosyan, Rouben Aroutiounian, Arsen Arakelyan

**Affiliations:** 1 Scientific Centre of Radiation Medicine and Burns, Ministry of Health, Yerevan, 0054, Armenia; 2 Institute of Molecular Biology, National Academy of Sciences, Yerevan, 0014, Armenia; 3 Yerevan State University, Ministry of Education and Science, Yerevan, 0025, Armenia

**Keywords:** Schiff base, L-tryptophan, copper (II) complex, synthesis, cytotoxicity, HeLa, KCL-22

## Abstract

Schiff bases and their metal-complexes are versatile compounds exhibiting a broad range of biological activities and thus actively used in the drug development process. The aim of the present study was the synthesis and characterization of new Schiff bases and their copper (II) complexes, derived from L-tryptophan and isomeric (2-; 3-; 4-) pyridinecarboxaldehydes, as well as the assessment of their toxicity *in vitro*. The optimal conditions of the Schiff base synthesis resulting in up to 75-85% yield of target products were identified. The structure-activity relationship analysis indicated that the location of the carboxaldehyde group at 2-, 3- or 4-position with regard to nitrogen of the pyridine ring in aldehyde component of the L-tryptophan derivative Schiff bases and corresponding copper complexes essentially change the biological activity of the compounds. The carboxaldehyde group at 2- and 4-positions leads to the higher cytotoxic activity, than that of at 3-position, and the presence of the copper in the complexes increases the cytotoxicity. Based on toxicity classification data, the compounds with non-toxic profile were identified, which can be used as new entities in the drug development process using Schiff base scaffold.

## Introduction

Schiff bases are considered as a very important class of organic ligands having a wide range of applications in many fields of biomedicine ^1– 3^. They are the condensation products of an amino compound with an active carbonyl compound and carry imine or azomethine (–C=N–) functional group, which is essential for their biological activity. Structurally, Schiff base is a nitrogen analog of an aldehyde or ketone, in which the carbonyl group (C=O) has been replaced by an imine or azomethine group. While aliphatic aldehyde containing Schiff bases are unstable in nature and readily get polymerized, aromatic aldehyde containing compounds are more stable due to conjugation system ^4^.

Schiff bases derived from aromatic aldehydes and aromatic amines are widely applicable in the fields of biology, inorganic and analytical chemistry ^5, 6^. Their biological activities are based on the earlier detected anti-inflammatory, antiviral, antibacterial, antifungal, antimalarial and antipyretic properties ^7– 10^. The bonding interactions between aromatic amino acid side chains of the receptor and aromatic/heteroaromatic rings of the ligand were revealed in most of X-ray crystal structures of protein complexes with small molecules. This protein-ligand recognition, based on aromatic ring involved non-covalent interactions, can ensure the application of Schiff bases, derived from aromatic aldehydes and amines, in drug design process ^11– 13^. Moreover, the evaluation of the structure-activity relationship of Schiff bases, derived from different substituted aromatic amines and aldehydes, demonstrated the importance of the latter for desired biological activity ^14– 15^.

Pyridinecarboxaldehyde derivatives of Schiff bases are of great interest because of their role in natural and synthetic organic chemistry. It is known, that pyridoxal-amino acid systems are important in numerous metabolic reactions intermediated with amino acid and pyridoxal. So far, pyridinecarboxaldehyde isomers characterized by different localization of carboxaldehyde group (2-, 3- or 4-) relative to nitrogen atom in pyridine ring are valuable precursors for complex forming Schiff bases, since they can exhibit physiological effects similar to pyridoxal-amino acid systems. Thus, the pyridinecarboxaldehydes containing Schiff bases are expected to have enhanced biological activities.

It is known that the binding of bioorganic molecules or drugs to metal ions drastically change their biomimetic properties, therapeutic effects, and pharmacological properties ^16^. Schiff base derivatives of aromatic amino acids are good chelating agents and capable to form stable complexes with transition metals and exhibit significant biological and enzymatic activities ^17, 18^. The most widely studied cation in this respect is copper, which is implicated in a wide range of vital cell functions. Copper has been proven to be beneficial against several diseases such as tuberculosis, rheumatoid, gastric ulcers and cancers ^19– 21^. Many non-toxic, lipid-soluble, small molecular mass copper chelate complexes have been shown to have superoxide dismutase- and catalase-like activities ^22– 24^, which makes them essential for *de novo* syntheses of metalloelement-dependent enzymes required for oxygen utilization and prevention of oxygen superoxide accumulation.

The present study describes the synthesis and characterization of isomeric 2-, 3- and 4-pyridinecarboxaldehydes and L-tryptophan derived Schiff bases and their copper (II) complexes, as well as the assessment of their cytotoxic activity.

## Methods

### Reagents

All chemicals and solvents used were of analytical grade. The reagents used for the synthesis of the Schiff bases and their copper (II) complexes were obtained from Sigma-Aldrich (Sigma-Aldrich Co. LLC, USA), including L-tryptophan, 2-; 3-; and 4-pyridinecarboxaldehydes, KOH, copper (II) acetate, and methanol.

### Chemical synthesis of Schiff bases derived from L-tryptophan and isomeric 2-, 3- and 4-pyridinecarboxaldehydes

Schiff bases ( **2pyr.Trp**, **3pyr.Trp**, and **4pyr.Trp**) were synthesized by condensation of potassium salt of L-tryptophan and isomeric (2-, 3-, 4-) pyridinecarboxaldehydes, respectively, in alcohol solutions (ethanol, methanol) in 5°C **–**25°C temperature range and 1:1 molar ratio. First, 10 mM of L-tryptophan was dissolved in 100 mL of alcohol solution, containing KOH (10mM) by permanent stirring under dry nitrogen at 18°C–20°C. Then 10 mM of the corresponding isomer of pyridincarboxyaldehyde (2-, 3- or 4-) was added to the resulting solution with stirring and refluxed at 50°C for 2 hours resulting in a yellow colored solution that indicates the Schiff base formation. The volume of the solution was then reduced *in vacuo* using a rotary evaporator. Anhydrous ether was added to deposit a yellowish precipitate, which was then re-crystallized from alcohol.

### Chemical synthesis of Schiff base copper (II) complexes

The obtained Schiff bases were served as ligands for the synthesis of appropriate copper (II) complexes (namely, Cu-2pyr.Trp, Cu-3pyr.Trp, and Cu-4pyr.Trp). The synthesis was performed at 20±2°С in alcohol media (methanol, ethanol) using potassium hydroxide and copper acetate. Complex formation was carried out in a reaction medium without preliminary isolation of Schiff bases. Compound isolation was performed by partial evaporation of the solvent, settling, centrifugation, re-crystallization, and vacuum drying.

### Characterization of Schiff bases and their copper (II) complexes

For characterization of Schiff bases and their copper (II) complexes the infrared (IR) absorbance spectra were obtained in the range of 4000–400 cm ^-1^s in Vaseline oil on KBr plates using Spectrometer IR 75 (Carl Zeiss, Jena). For assessment of thermal stability of obtained compounds the IR absorbance spectra were recorded every 30 minutes in the 60°С – 100 ^о^С temperature range for 2 hours.

The elemental analysis was performed by combustion in a pure oxygen environment using PerkinElmer 2400 Series II CHNS/O Elemental Analyzer (PerkinElmer, USA). The nuclear magnetic resonance (NMR) spectra were obtained in D _2_O and CD _3_OD on Spectrometer Varian 300 MHz (Agilent, USA).

### Determination of solubility of substances tested for biological studies

Solubility assessment of synthesized compounds was carried out according to standard test method protocol ^25^. Schiff bases were water-soluble, while their copper (II) complexes were soluble in dimethyl sulfoxide (DMSO). Stock solutions of Schiff bases and their copper (II) complexes at the concentration of 10 mM/mL were prepared and diluted with nutrition medium RPMI-1640 or DMEM. Only freshly prepared solutions were used in experiments.

### Cell cultures

Human HeLa (cervix carcinoma) and KCL-22 (chronic myeloid leukemia) cell lines were obtained from the cell culture collection of the Institute of Molecular Biology (Yerevan, Armenia). Growth media (DMEM and RPMI-1640) as well as media supplements were obtained from Sigma-Aldrich. The human HeLa and KCL-22 cell lines were routinely maintained at 37°C in the growth medium DMEM (HeLa) and RPMI-1640 (KCL-22), supplemented with 10% fetal bovine serum (HyClone, UK), 2 mM L-glutamine (Sigma Aldrich, Germany), 100 IU/mL penicillin (Sigma Aldrich, Germany) and 100 μg/mL streptomycin (Sigma Aldrich, Germany).

### The cytotoxicity assessment of Schiff bases and their copper (II) complexes

The cytotoxicity of test compounds was assessed using standard protocols for Trypan blue exclusion test and neutral red uptake (NRU) assay ^26, 27^.

*Trypan blue exclusion test:* The KCL-22 cells were seeded into 15 ml glass vials at the density of 0.5 × 10 ^6^ cells/mL. After 48 hours the test compounds were added at the concentrations of 0.1 µM/mL, 1 µM/mL, 10 µM/mL, 100 µM/mL, and 1000 µM/mL. After further incubation for 48 hours, cells were stained with 0.4% Trypan blue solution for 5-15 minutes and counted in a haemocytometer under a light microscope. The viable cell number was determined.

*NRU assay*: The HeLa cells were seeded at the density of 0.3 × 10 ^6^ cells/mL into 96-well plates (Corning, USA), incubated for 48 hours, and then test compounds were added to the cell cultures at the concentrations of 0.1 µM/mL, 1 µM/mL, 10 µM/mL, 100 µM/mL, and 1000 µM/mL. After further incubation for 48 hours the NRU assay was performed. The absorbance was measured using a microplate reader (Human Reader HS, Germany) at a wavelength of 570 nm.

Cell viability was expressed as a percentage of the negative control (cell cultures with no treatment). Doses inducing 50% inhibition of cell viability (the IC _50_ value) were calculated to determine the cytotoxicity of Schiff bases and their copper(II) complexes.

### Toxicity classification of Schiff bases and their copper (II) complexes

The extrapolation of obtained IC _50_ values into LD _50_ values for compound acute rodent oral toxicity *in vivo* was performed. The regression formula was used to weigh up the starting doses for single oral application in rats ^28^:

log⁡ LD50 (mMl/kg) =0.435 log⁡ IC50 (mM/L)+ 0.625

Based on obtained LD _50_ values the class of toxicity was assigned to all synthesized compounds ^29^. Since the human cell lines were used for the experiments, the IC _50_ values obtained have also prognostic significance for human ^30^.

### Statistical analysis

All experiments were done in at least three replicates. At least triplicate cultures were scored for an experimental point. All values were expressed as means ± SE. The Student’s one tailed t-test was applied for statistical analysis of results, p < 0.05 was considered as the statistically significant.

## Results and Discussion

### Synthesis and characterization of Schiff bases and their copper (II) complexes

The optimal conditions of the Schiff base synthesis with the use of potassium salt of L-tryptophan were identified, allowing to obtain the yield of target products up to 85% for 2pyr.Trp, 75% for 3pyr.Trp, and 80% for 4pyr.Trp. Based on ^1^H NMR spectra inspection for synthesized Schiff bases in D _2_O and CD _3_OD, the loss of 2 singlet signals of СН2 group was revealed, confirming the presence of target compounds. The observed ^1^Н NMR spectral data, particularly, the presence of CH signal (duplet-duplets) of –СН2–СН– in the range of 4.1–4.5 ppm are characteristic for Schiff bases. The results of elemental analysis of 2pyr.Trp, 3pyr.Trp and 4pyr.Trp Schiff bases are presented in the Table 1.

**Table 1.** The elemental analysis of synthesized Schiff bases.

Schiff base	K, %		C, %		H, %		N, %	
	Calculated	Observed	Calculated	Observed	Calculated	Observed	Calculated	Observed
K.2pyr.Trp	11.80	11.62	61.61	61.88	4.26	4.63	12.68	12. 53
K.3pyr.Trp	11.80	11.58	61.61	61.79	4.26	4.52	12.68	12.93
K.4pyr.Trp	11.80	11.67	61.61	61.92	4.26	4.58	12.68	12.85

**Figure 1.**
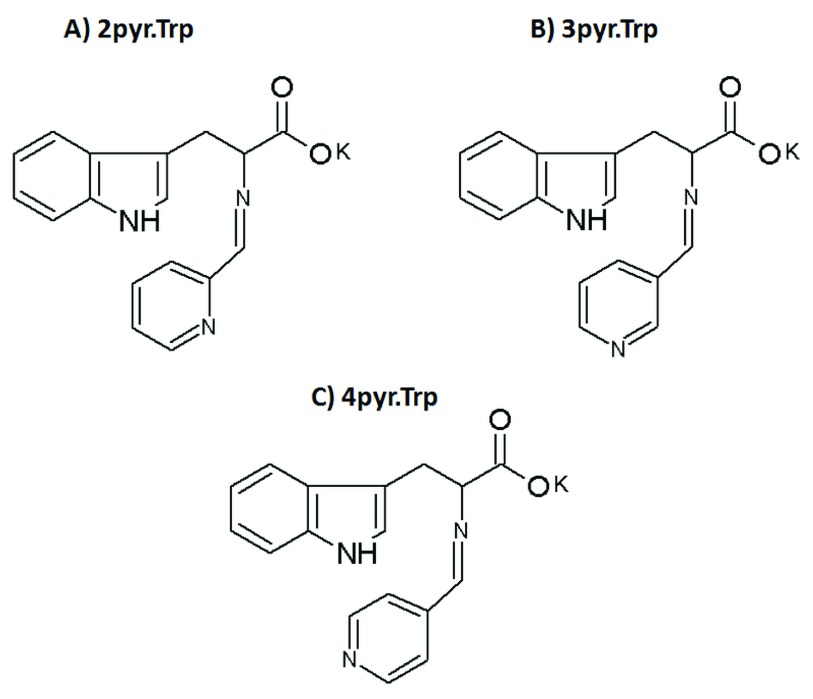
Suggested structure of the 2pyr.Trp ( **A**), 3pyr.Trp ( **B**) and 4pyr.Trp ( **C**) Schiff bases.

The brutto chemical formula for **2pyr.Trp**, **3pyr.Trp**and **4pyr.Trp** Schiff bases was identified as C _17_H _14_N _3_O _2_K (Mr=331.41). The suggested structure of synthesized Schiff bases is presented in the Figure 1. Decomposition temperature of **2pyr.Trp**, **3pyr.Trp**, and **4pyr.Trp** Schiff bases was in the range of 180ºC – 190ºC.

**Figure 2.**
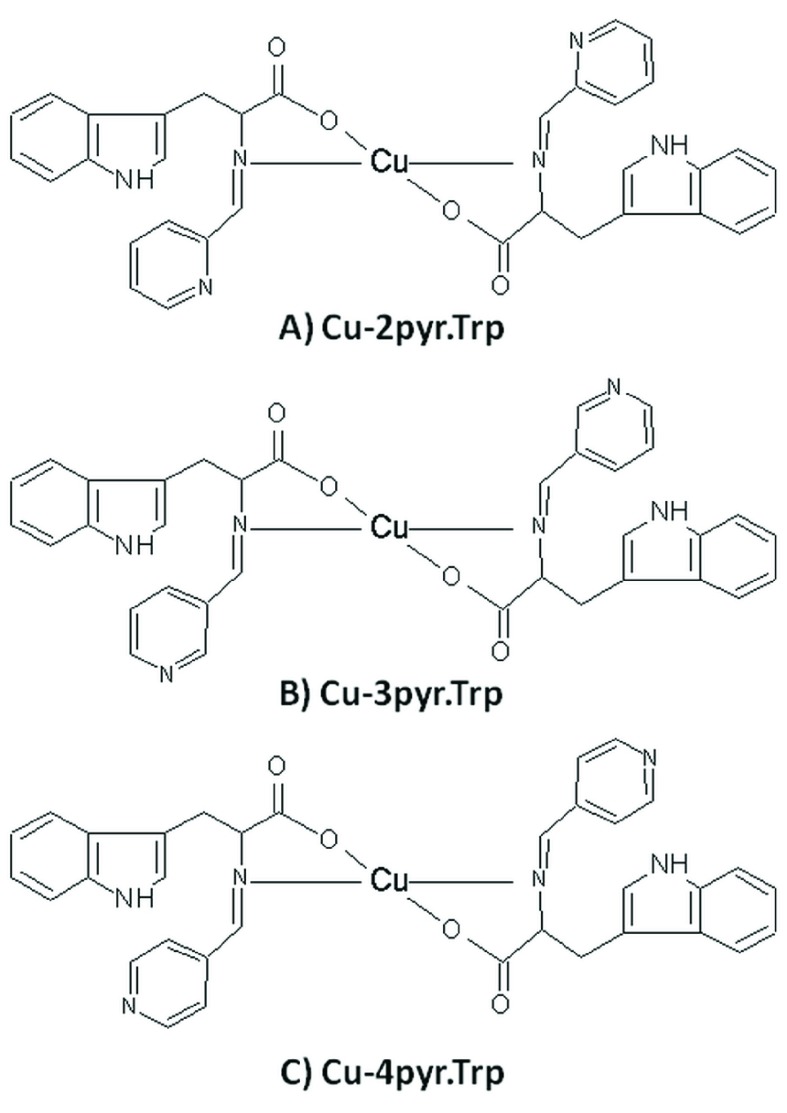
Suggested structure of Cu-2pyr.Trp ( **A**), Cu-3pyr.Trp ( **B**), Cu-4pyr.Trp ( **C**) metallocomplexes.

Obtained Schiff bases were then used for the synthesis of corresponding copper (II) complexes ( **Cu-2pyr.Trp**, **Cu-3pyr.Trp**, **Cu-4pyr.Trp**). Based on IR analysis the shift of IR absorbance bands upon formation of copper metallocomplexes with Schiff bases was observed ( Table 2). Upon formation of Schiff bases the valence deviation (NH) of the tryptophan indole ring was shifted from 3430cm ^-1^ to 3180-3190cm ^-1^ due to the intramolecular interaction of indole and pyridine rings. In copper metallocomplexes this band appeared in the area of 3250–3270cm ^-1^, which was associated with the changes in conjugation linkage degree (C=N) with pyridine ring caused by coordination bond Cu....N. This in turn resulted in changes of interactions between the pyridine and indole rings. Coordination bond Cu....N caused also a shift of valence deviations (C=N) towards low frequencies, and this band was practically overlapped by the band of valence deviations of (C=О ^-^). The results of the determination of the copper content in metallocomplexes by atomic absorption and elemental analysis of carbon, nitrogen and hydrogen are presented in the Table 3. The obtained data allowed to suggest that metallocomplexes contain two Schiff base ligands and have brutto formula C _34_H _28_N _6_O _4_Cu (Mr = 648.17). The inferred structures of **Cu-2pyr.Trp, Cu-3pyr.Trp, Cu-4pyr.Trp** metallocomplexes are presented in the Figure 2. Thermostability assessment demonstrated no changes in IR spectra at 100 ^о^С during 2 hours. Decomposition temperature for these compounds was at a range of 180 ^о^C – 190 ^о^C without melting.

**Table 2.** The IR absorbance spectra of Schiff bases derived from L-tryptophan and their copper complexes.

Valence deviations	Schiff bases	Metallocomplexes
ν (NH), cm ^-1^	3180 – 3190	3250 – 3270
ν (C=N), cm ^-1^	1625 – 1645	1611 – 1625
ν (C=О), cm ^-1^	1583 – 1595	
ν (C-N), cm ^-1^	1080 – 1088	1060 – 1080
ν (C-O), cm ^-1^	1103 – 1109	1106 – 1109
ν (C-N), cm ^-1^	1080 – 1088	1060 – 1080
ν (C-C), cm ^-1^	1013 – 1016	1013 – 1020

**Table 3.** Elemental analysis of Cu-2pyr.Trp, Cu-3pyr.Trp and Cu-4pyr.Trp metallocomplexes.

Cu(II) complex	C, %		H, %		Cu, %		N, %	
	Calculated	Observed	Calculated	Observed	Calculated	Observed	Calculated	Observed
Cu-2pyr.Trp	63.00	63.28	4.35	4.76	9.80	9.34	12.97	12.81
Cu-3pyr.Trp	63.00	63.48	4.35	4.81	9.80	10.31	12.97	13.19
Cu-4pyr.Trp	63.00	62.67	4.36	4.64	9.80	10.33	12.97	12.62

### The cytotoxicity of Schiff bases and their copper (II) complexes

**Figure 3.**
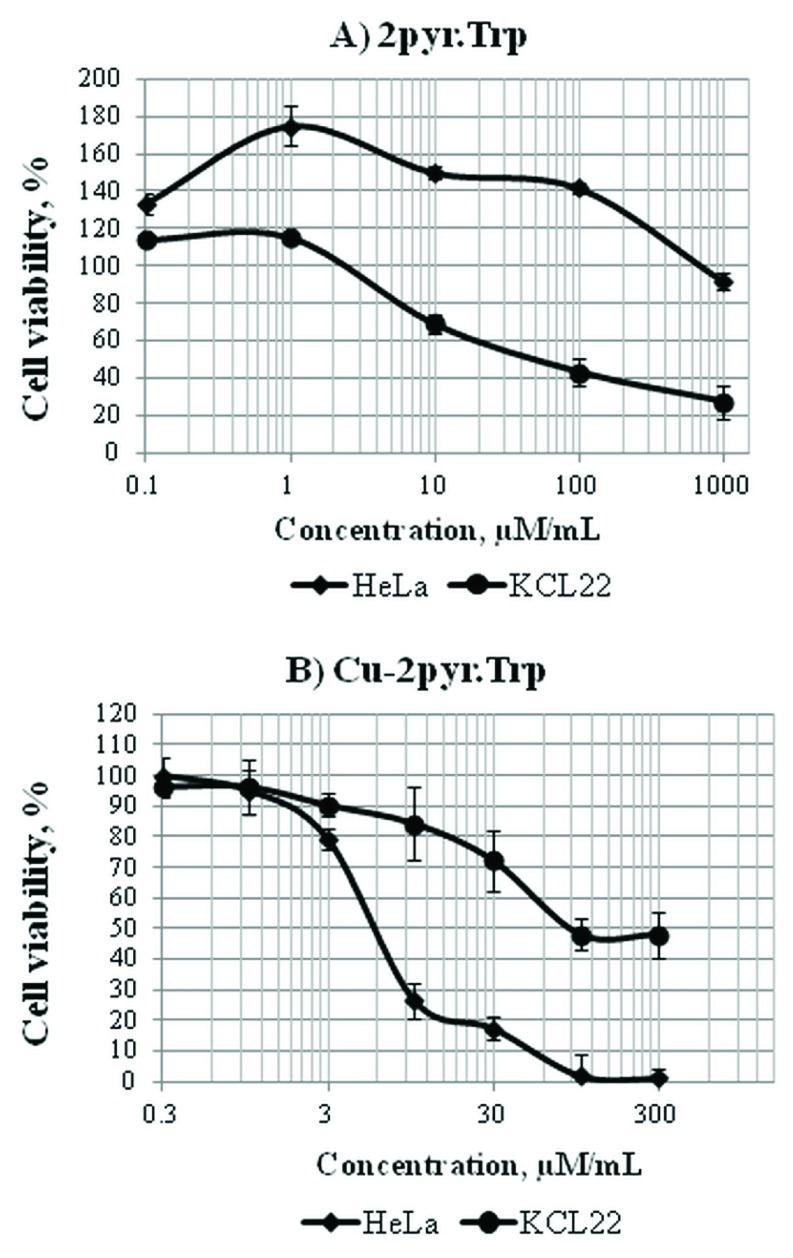
The cytotoxicity of 2pyr.Trp ( **A**) and Cu-2pyr.Trp ( **B**) in HeLa and KCL-22 cell lines. Dose-response curves were obtained after 48 hours of treatment with Schiff base 2pyr.Trp and its copper(II) complex Cu-2pyr.Trp at the concentration range of 0.1–1000 µM/mL. Cell viability was expressed as a percentage of the negative control (cell cultures with no treatment). Doses inducing 50% inhibition of cell viability (the IC _50_ value) were calculated to determine the cytotoxicity of 2pyr.Trp and Cu-2pyr.Trp. The IC _50_ value estimated for 2pyr.Trp in KCL-22 cell line was equal to 56±9.1 μM/mL, whereas the viability of HeLa cells was more than 90% at the highest concentration tested ( **A**). The IC _50_ values estimated for Cu-2pyr.Trp were equal to 7±1.7 μM/mL and 80±7.5 μM/mL for HeLa and KCL-22 cell lines, respectively ( **B**).

The synthesized Schiff bases and their copper complexes were tested *in vitro* to determine their cytotoxicity in Hela and KCL-22 cell lines. Our results indicate that the cytotoxic activity of **2pyr.Trp** and its copper (II) complex depends on a cell line ( Figure 3, A and B, Dataset 1, **File 1**). In case of **2pyr.Trp** action in HeLa cell line, a hormesis effect was apparent, since a significant increase in the cell number was observed at lower concentrations of 0.1-1µM/mL. Further dosage increase, however, did not lead to the total cell death, since the cell viability was more than 90% compared to untreated cells, even at the highest concentration tested (1000 µM/mL). In contrast, the KCL-22 cell line was more sensitive against cytotoxicity of **2pyr.Trp**; the hormesis effect was only slightly visible, but the further dose-dependent cell viability decrease was observed and resulting in 20% of cell viability at the highest tested concentration (1000 µM/mL) ( Figure 3, A). In case of **Cu-2pyr.Trp** the sensitivity of cell lines was reversed. Starting from 10 µM/mL concentration the viability of HeLa cells decreased substantially compared with the KCL-22 cell line ( Figure 3, B).

**Figure 4.**
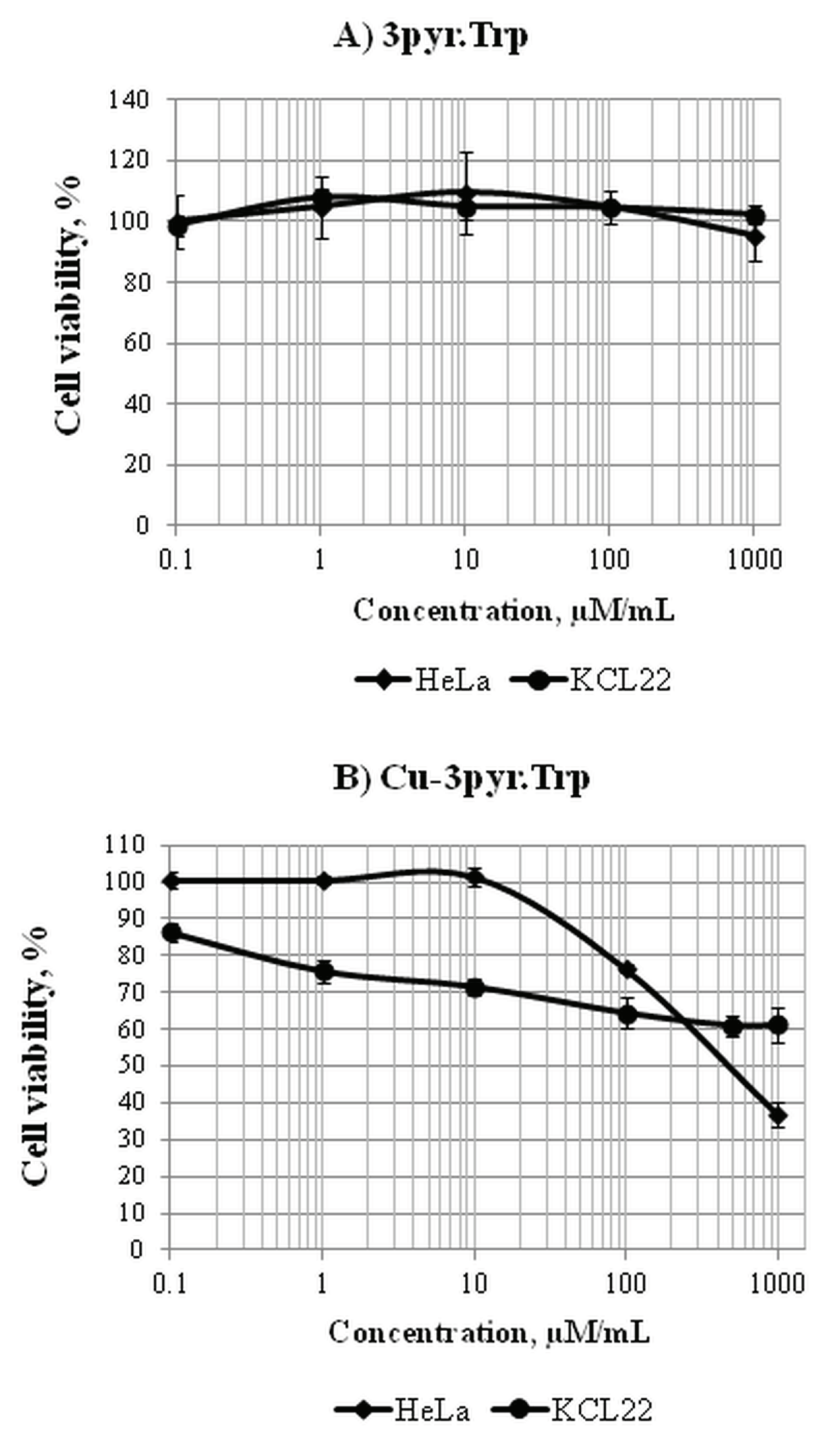
The cytotoxicity of 3pyr.Trp ( **A**) and Cu-3pyr.Trp ( **B**) in HeLa and KCL-22 cell lines. Dose-response curves were obtained after 48 hours of treatment with Schiff base 3pyr.Trp and its copper(II) complex Cu-3pyr.Trp at the concentration range of 0.1–1000 µM/mL. Cell viability was expressed as a percentage of the negative control (cell cultures with no treatment). Doses inducing 50% inhibition of cell viability (the IC _50_ value) were calculated to determine the cytotoxicity of 3pyr.Trp and Cu-3pyr.Trp. The non-cytotoxic profile was observed for 3pyr.Trp in both cell lines, since the viability of HeLa and KCL-22 cells was around 100% at the highest concentration tested ( **A**). The Cu-3pyr.Trp demonstrated the increased cytotoxic activity against both cell lines, however, the IC _50_ value was possible to estimate only for HeLa cells (500±5.6 μM/mL), since the viability of KCL-22 cells was more than 60% at the highest concentration tested ( **B**).

The non-cytotoxic profile was observed for Schiff base **3pyr.Trp** in both cell lines ( Figure 4, A, Dataset 1, **File 2**), while, **Cu-3pyr.Trp** demonstrated the increased cytotoxic activity against both cell lines. ( Figure 4, B). However, the IC _50_ value was possible to estimate only for HeLa cells, since the viability of KCL-22 cells was more than 60% at the highest concentration tested (1000 µM/mL).

**Figure 5.**
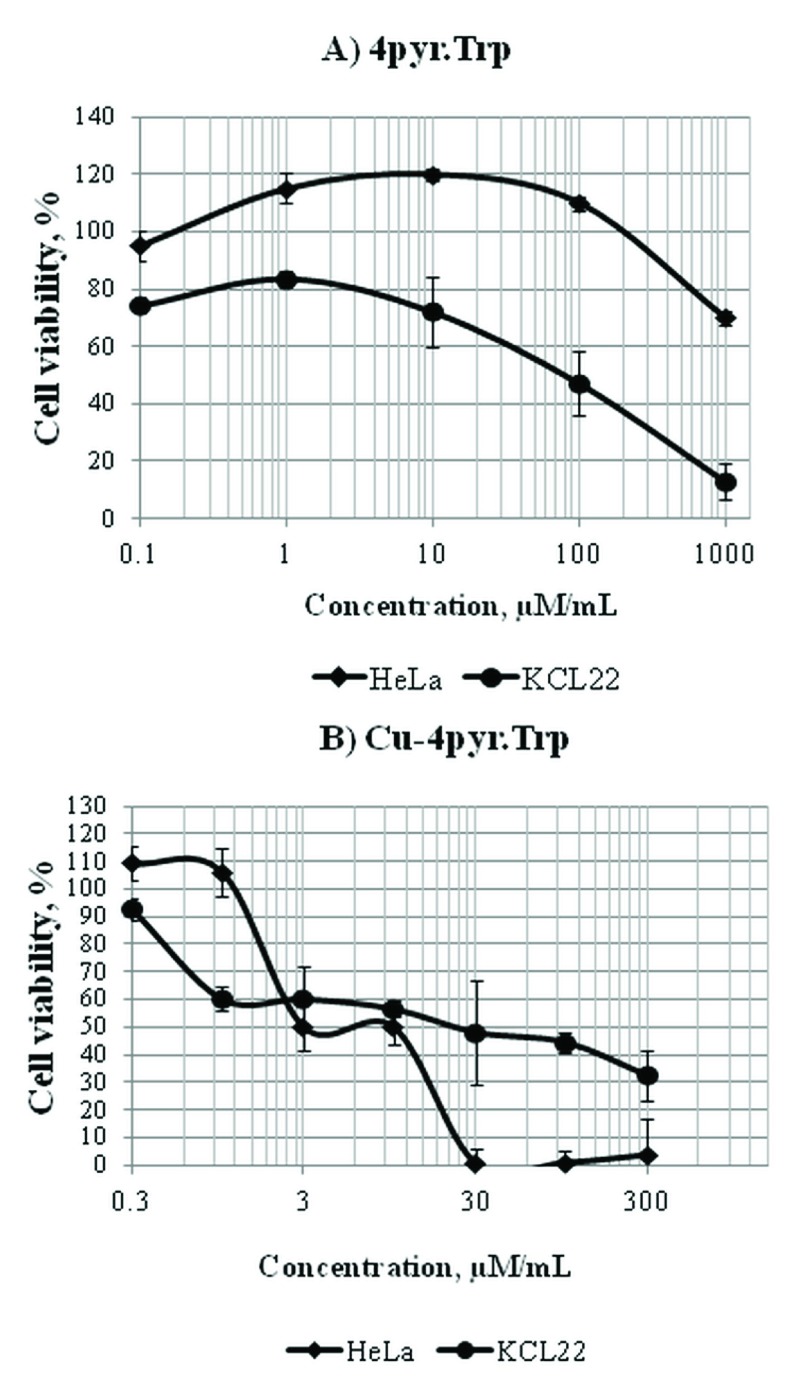
The cytotoxicity of 4pyr.Trp ( **A**) and Cu-4pyr.Trp ( **B**) in HeLa and KCL-22 cell lines. Dose-response curves were obtained after 48 hours of treatment with Schiff base 4pyr.Trp and its copper(II) complex Cu-4pyr.Trp at the concentration range of 0.1–1000 µM/mL. Cell viability was expressed as a percentage of the negative control (cell cultures with no treatment). Doses inducing 50% inhibition of cell viability (the IC _50_ value) were calculated to determine the cytotoxicity of 4pyr.Trp and Cu-4pyr.Trp. The IC _50_ value estimated for 4pyr.Trp in KCL-22 cell line was equal to 100±6.5 μM/mL, whereas the viability of HeLa cells was more than 60% at the highest concentration tested ( **A**). The IC _50_ values estimated for Cu-4pyr.Trp were equal to 10±5 μM/mL and 30±3.8 μM/mL for HeLa and KCL-22 cell lines, respectively ( **B**).

The toxicity profile of **4pyr.Trp** ( Figure 5, A and B, Dataset 1, **F3**) was similar to **2pyr.Trp**, since the slight hormesis effect was again evident in HeLa cell line, while the KCL-22 cells were more sensitive against its cytotoxic activity ( Figure 5, A). Despite these similarities, the overall cytotoxic activity of **4pyr.Trp** was higher compared to **2pyr.Trp.** The level of cell viability at the highest tested concentration (1000 µM/mL) for **4pyr.Trp** ( Figure 5, A) were 70% for HeLa and 12% for KCL-22, respectively, while in case of **2pyr.Trp** viability levels were 90% (HeLa) and 30% (KCL-22), respectively ( Figure 4, A). Again, HeLa cells were more susceptible to the cytotoxicity of **Cu-4pyr.Trp** than KCL-22 cells ( Figure 5, B). Earlier, several Schiff bases were tested *in vitro* for their cytotoxic activity against different cell lines and the structure activity relationship of compounds was discussed ^17, 31, 32^. Furthermore, Kril *et al*. reported on the hormesis effect for MCF-7 and 647-V tumour cells, and suggested that receptor-mediated mechanisms are responsible for the observed phenomenon. Here, we also demonstrated the hormesis effect in HeLa cell line, which supports the statement about potential receptor-binding ability of Schiff bases due to the presence of carbon–nitrogen double bond. The changes in cytotoxic activity against cancer cell lines were shown earlier depending on the presence of different groups (chloro, methoxy, nitro, and phenyl) in aromatic rings of a Schiff base molecule ^32^. Furthermore, we have noted that even the localization of carboxaldehyde group at 2-, 3- or 4-position with regard to nitrogen of aromatic ring can affect the cytotoxicity of Schiff bases.

Based on dose-response curves the half-maximal inhibitory concentrations (IC _50_ values) were estimated for Schiff bases and their copper (II) complexes ( Table 4). Our data suggest that Schiff bases **2pyr.Trp, 3pyr.Trp** and **4pyr.Trp** are non-toxic for HeLa cells since the IC _50_ values were impossible to estimate even at the highest tested concentration (1000 µM/mL). The **2pyr.Trp** (IC _50_=56±9.1 μM/mL) was two times more toxic against the KCL-22 cell line, than **4pyr.Trp** (IC _50_=100±6.5 μM/mL), while the **3pyr.Trp** demonstrated the same non-toxic profile as it was shown in HeLa cells.

**Table 4.** The cytotoxicity (expressed as IC _50_, μM/mL) of tested compounds in HeLa and KCL-22 cells.

Cells lines	L-tryptophan Schiff bases and their copper(II) complexes					
	2pyr.Trp	3pyr.Trp	4pyr.Trp	Cu-2pyr.Trp	Cu-3pyr.Trp	Cu-4pyr.Trp
HeLa	> 1000	> 1000	> 1000	7±1.7	500±5.6	10±5
KCL-22	56±9.1	> 1000	100±6.5	80±7.5	>1000	30±3.8
**LD _50_,** mg */*kg (extrapolated)	>4000	>5000	>5000	>2000	>5000	>2000
**Toxicity classification**	Class III Slightly toxic	Class IV Non-toxic	Class IV Non-toxic	Class III Slightly toxic	Class IV Non-toxic	Class III Slightly toxic

**Figure 6.**
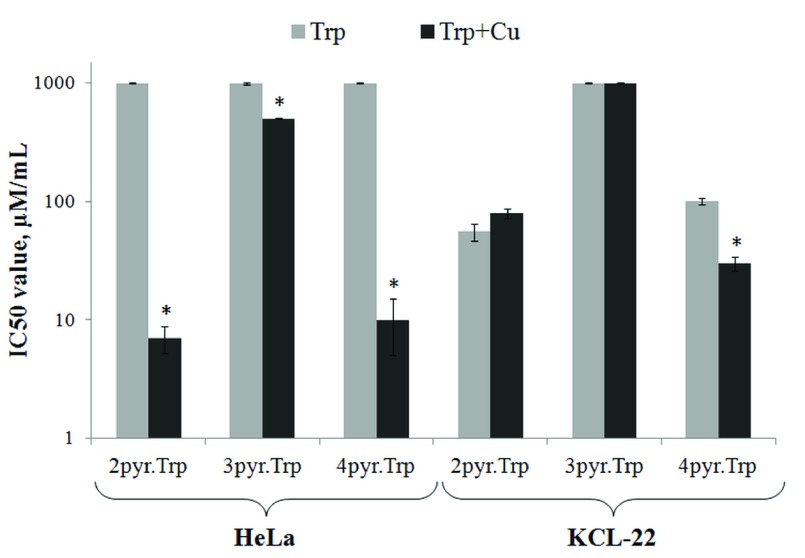
Comparison of Schiff bases and copper(II) complexes cytotoxicity in HeLa and KCL-22 cell lines. *p<0.01.

The cytotoxic activity was observed for **Cu-2pyr.Trp, Cu-3pyr.Trp and Cu-4pyr.Trp** in HeLa cell line with the IC _50_ values of 7±1.7 μM/mL, 500±5.6 μM/mL and 10±5.0 μM/mL, respectively. Those IC _50_ values were significantly lower in comparison with their copper free analogs. The same tendency was demonstrated for **Cu-4pyr.Trp** in KCL-22 cell line, where the IC _50_ value decreased up to 30±3.7 μM/mL. In case of **Cu-2pyr.Trp** and **Cu-3pyr.Trp** complexes tested in KCL-22 cell line, no significant differences in IC _50_ values were observed in comparison with their copper free analogs. Thus, it can be assumed that the cytotoxic activity of Schiff bases tends to increase at complex formation with the copper molecule ( Figure 6).

Testing of the compounds’ effects on the viability of cells grown in culture is widely used as a predictor of potential toxic effects in whole animals ^28^. Our extrapolated data on the predicted LD _50_ doses demonstrated that the tested compounds **3pyr.Trp**, **4pyr.Trp**, and **Cu-3pyr.Trp** belong to the Class IV of non-toxic chemicals, while **2pyr.Trp**, **Cu-2pyr.Trp**, and **Cu-4pyr.Trp** belong to the Class III of slightly toxic compounds ( Table 4) ^29^. Since the human cell lines were used for the experiments, those hazard classification data have also a prognostic significance for the human. The United States Food and Drug Administration (FDA) states that it is essential to perform toxicological studies during the development of new drugs, since the desirable pharmacological activity needs to be achieved in the absence of acute toxicity ^33^. The non-toxic profile of **3pyr.Trp**, **4pyr.Trp** and **Cu-3pyr.Trp** Schiff bases indicates that this compounds can be considered as new entities in drug development process.

Raw data of generated dose-response curves The raw data of all generated dose-response for 2pyr.Trp and Cu-2pyr.Trp are provided. The readme file contains descriptions for each data file. Click here for additional data file. Copyright: © 2016 Malakyan M et al. 2016 Data associated with the article are available under the terms of the Creative Commons Zero "No rights reserved" data waiver (CC0 1.0 Public domain dedication).

## Conclusions

We have synthesized and characterized several new Schiff bases of aromatic amino acid derivatives and their copper complexes. Cytotoxicity tests indicated that **3pyr.Trp**, **4pyr.Trp**, and **Cu-3pyr.Trp** are non-toxic for human, whereas compounds **2pyr.Trp**, **Cu-2pyr.Trp**, and **Cu-4pyr.Trp** retain slight toxicity. Moreover, obtained results indicate that cell lines HeLa (epithelial origin) and KCL-22 (derived from blood) vary in sensitivity to the cytotoxic action of the tested compounds; the latter suggests the tissue-/cell line-specificity of their effect. The results also demonstrate that structural alterations, namely, the localization of the carboxaldehyde group at 2-, 3- or 4-position with regard to nitrogen of pyridine ring in aldehyde component of the L-tryptophan derivative Schiff bases and corresponding copper complexes essentially change the biological activity of the compounds tested. The carboxaldehyde group at 2- and 4-positions leads to the higher cytotoxic activity, than that of at 3-position, the presence of the copper in the complexes, mostly increases the cytotoxicity. Thus, the results obtained may be used for the further development of pharmaceutical agents based on L-tryptophan and pyridinecarboxaldehyde derived Schiff bases and their copper(II) complexes.

## Data availability

The data referenced by this article are under copyright with the following copyright statement: Copyright: © 2016 Malakyan M et al.

Data associated with the article are available under the terms of the Creative Commons Zero "No rights reserved" data waiver (CC0 1.0 Public domain dedication). 

F1000Research: Dataset 1. raw data of generated dose-response curves, 10.5256/f1000research.9226.d130235 ^34^
