# Supplementary material for: Synthesis, characterization and toxicity studies of pyridinecarboxaldehydes and L-tryptophan derived Schiff bases and corresponding copper (II) complexes
Source: F1000Res. 2016 Aug 5;5:1921. [Version 1] doi: 10.12688/f1000research.9226.1 (PMC5333612; doi:10.12688/f1000research.9226.1)
Supplement: Peer review history

# Peer review: Referee response for version 1

**DOI:** 10.5256/f1000research.9930.r17256

**Published:** 2016-11-04

**Authors:** Salvatore Failla, Ivan Pietro Oliveri

**Affiliations:** 1 Department of Chemical Sciences, University of Catania, Catania, Italy

The focuses of the paper of Malakyan, Arakelyan and co-workers are the synthesis and characterization of isomeric 2-, 3- and 4-pyridinecarboxaldehydes and L-tryptophan derived Schiff bases and their copper (II) complexes and the study of their cytotoxic activity respect to two different cell lines, in particular the Human HeLa (cervix carcinoma) and the KCL-22 (chronic myeloid leukemia).

 The paper is well written, clear and highlights the differences between the cytotoxic activity of all Schiff base ligands and relative copper (II) complexes investigated through toxicity profiles. Moreover, the characterization of all compounds involved is detailed described and, in general, we find the results achieved from this investigation interesting.

 We suggest discussing two points to improve the quality of paper:

1. How metal complexation influences the cytotoxic activity of **Cu-2pyr.Trp**, **Cu-3pyr.Trp** and **Cu-4pyr.Trp** respect to relative Schiff base ligands.
2. The effect of the localization of carboxaldehyde group at 2-, 3- or 4-position with regard to nitrogen of aromatic ring on cytotoxic activity of all compound investigated.

 

 Finally, a minor revision. In the first paragraph of the Results and Discussion section, especially in the “Synthesis and characterization of Schiff bases and their copper (II) complexes” section, the sentence “ *Based on 1H NMR spectra inspection for synthesized Schiff bases in D2O and CD3OD, the loss of 2 singlet signals of CH2 group was revealed, confirming the presence of target compounds.*” seems in contrast with the next one. This sentence should be reworked considering that the C *H*=N ^1^H NMR signal is diagnostic for evaluation of the Schiff bases formation.

We have read this submission. We believe that we have an appropriate level of expertise to confirm that it is of an acceptable scientific standard.

# Peer review: Referee response for version 1

**DOI:** 10.5256/f1000research.9930.r16481

**Published:** 2016-09-21

**Authors:** Carmel E. Mothersill

**Affiliations:** 1 Department of Medical Physics and Applied Radiation Sciences, McMaster University, Hamilton, ON, Canada

I believe that the title is appropriate for the content of this article and the abstract represents an excellent summary of the work. This very interesting paper describes the synthesis and cytotoxicity testing of several Schiff bases with potential use in cancer therapy. The bases are characterised and then tested for toxicity to human cell lines using two well established tests. The results reveal differences in responses of the two cell lines (one blood and one epithhelial derived) making them potentially very useful. The authors also hint at different biological mechanisms being involved.

 The structure, methods and analysis of the results from the study are very well designed. I think the conclusions are sensible, balanced and justified on the basis of the results of the study. There is enough information provided to be able to replicate the experiment and the data are in a usable format/ structure and have all been provided.

 In summary, the paper is well written and the data appear sound.

I have read this submission. I believe that I have an appropriate level of expertise to confirm that it is of an acceptable scientific standard.
